# Dilated Cardiomyopathy May Be Associated With a Novel Mitochondrial tRNA^Ser(AGY)^ Mutation

**DOI:** 10.1155/humu/7888334

**Published:** 2025-06-06

**Authors:** Yu Ding, Xuejiao Yu, Jian Xu, Shunrong Zhang, Jianhang Leng

**Affiliations:** ^1^Department of Clinical Laboratory, Hangzhou First People's Hospital, Hangzhou, Zhejiang, China; ^2^Department of Clinical Laboratory, Quzhou People's Hospital, Quzhou, Zhejiang, China; ^3^Department of Geriatrics, Hangzhou First People's Hospital, Hangzhou, Zhejiang, China

**Keywords:** dilated cardiomyopathy, mitochondrial dysfunction, mt-tRNA^Cys^ 5821G>A, mt-tRNA^Ser(AGY)^ 12265A>G, mutations

## Abstract

Dilated cardiomyopathy (DCM) is a serious public health problem that increases the risk of developing heart failure. Most recently, increasing evidence has shown that mitochondrial dysfunction caused by mitochondrial tRNA (mt-tRNA) mutations plays a putative role in the pathogenesis of this disease, despite its pathophysiology remaining poorly understood. In this study, a novel 12265A>G mutation in mt-tRNA^Ser(AGY)^ was identified from a Chinese pedigree with maternally inherited DCM, together with a known mt-tRNA^Cys^ 5821G>A mutation. Interestingly, the novel m.12265A>G mutation changed the well-conserved adenosine at Position 73 (A73) to guanine (G73) at the 3⁣′-end of the mt-tRNA^Ser(AGY)^ acceptor arm, while the G-to-A transition at 5821 occurred at the acceptor arm of mt-tRNA^Cys^, disrupting conserved base pairing (G6-C67). Transmitochondrial cybrid-based study demonstrated that cell lines with m.12265A>G and m.5821G>A mutations showed impaired mitochondrial functions, including significant reductions in mitochondrial ATP, membrane potential, NAD^+^/NADH ratio, mitochondrial DNA (mtDNA) content, mitochondrial transcription factor A (TFAM) mRNA expression levels, and respiratory chain enzyme Complex I and III activities, whereas the levels of reactive oxygen species (ROS), calcium ions (Ca^2+^), and lactate were enhanced in mutant cells compared to controls (*p* < 0.05). Thus, the m.12265A>G and m.5821G>A mutations may affect mt-tRNA metabolism and impair mitochondrial function, which is involved in DCM. Taken together, our study broadens the genotypic interpretation of mt-tRNA mutations linked to disease.

## 1. Introduction

Cardiomyopathy is one of the most common cardiovascular disorders, characterized by an abnormality in heart structure and function. There are four types of cardiomyopathies, called hypertrophic cardiomyopathy (HCM), dilated cardiomyopathy (DCM), arrhythmogenic cardiomyopathy (AC), and left ventricular noncompaction (LVNC) [1]. Among these, DCM is the most frequently observed heart disease featured by cardiac dilatation and impairments of the left ventricle (LV) [2]. Globally, the incidence of DCM is 1:2700, and familial DCM patients are found to account for approximately 30%–50% of cases. To date, 70 loci which mapped to 63 prioritized genes have been reported to be associated with this disease [3]. Nevertheless, DCM is often misdiagnosed due to its complex clinical phenotypes. Several case–control studies have suggested that mutations in *BAG3* [4], *C10orf71* [5], *TNNI3* [6], and *CASZ1* [7] are associated with DCM. However, the molecular mechanisms that trigger DCM development or diagnosis remain poorly understood.

The heart contains abundant mitochondria, which occupy approximately 40% volume of the adult cardiomyocytes [8]. Mitochondrial DNA (mtDNA) is a 16,569 bp molecule, containing 37 genes encoded for 13 proteins, 22 tRNAs, and two rRNAs which are important for electron transport chain (ETC) functions [9]. Although mt-tRNAs occupy only ~5% of the whole mtDNA genes, 70%~75% of the reported pathogenic mitochondrial mutations are localized at these regions, highlighting the importance of mt-tRNA mutations to mitochondrial translation and oxidative phosphorylation (OXPHOS) [10]. Mutations in mt-tRNA genes are proposed to impair 5⁣′ or 3⁣′-end processing and chemical modifications and disrupt mitochondrial function [11].

Most recently, to explore the potential associations between mt-tRNA mutations and DCM, a total of 180 DCM subjects and 420 healthy individuals undergo the mutational analysis of entire mt-tRNA genes via Hangzhou First People's Hospital; after genetic counseling and mtDNA sequence analysis, we identified a novel mt-tRNA^Ser(AGY)^ 12265A>G mutation and known mt-tRNA^Cys^ 5821G>A mutation from a maternally inherited DCM pedigree. The pathogenic roles of the m.12265A>G and m.5821G>A mutations were further verified by determining the mitochondrial functions in cybrid cells with and without these mutations.

## 2. Materials and Methods

### 2.1. Participants

**Figure 1:**
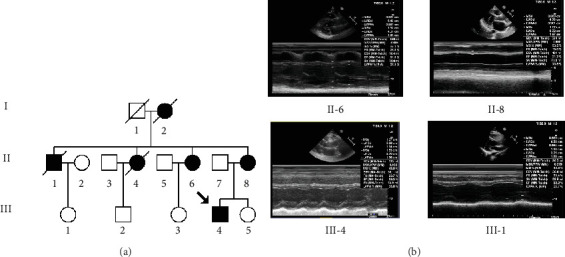
Clinical presentation of one Han Chinese pedigree with DCM. (a) One three-generation Han Chinese family with maternally inherited DCM; arrow denotes the proband; patients are indicated by filled symbols. (b) Clinical examination of matrilineal relatives suffering from DCM by using two-dimensional echocardiogram.

As part of the genetic screening program for DCM-related mt-tRNA mutations, one Han Chinese pedigree (Figure 1a) was ascertained via the Department of Geriatrics, Hangzhou First People's Hospital. We first obtained the informed consent and blood samples from each individual participating in this study. The current study was approved by the Ethics Committee of Hangzhou First People's Hospital (Approval Number KY-20240408-0123-01). We next performed a questionnaire for all matrilineal relatives from this pedigree to record the family history of DCM, as well as other clinical disorders. In addition, 420 healthy subjects (including 200 males and 220 females, aged 34~50 years, with an average age of 42 years) were recruited from a panel of unaffected, genetically unrelated individuals from Quzhou and Hangzhou cities in Zhejiang Province as controls.

### 2.2. Clinical Evaluations

All matrilineal relatives within the pedigree underwent a comprehensive clinical evaluation including physical examination, M-mode and two-dimensional echocardiography (ECHO), and electrocardiography (ECG), which was conducted as described previously [12].

DCM can be diagnosed as follows: (1) fractional shortening (FS) < 25% and/or ejection fraction < 45% and (2) left ventricular end diastolic diameter (LVEDd) > 117%, excluding any known cause of myocardial disease [13].

### 2.3. Mutation Analysis

Genomic DNA was extracted from venous blood of family members (III-4, II-6, and II-8) using the Blood Genome DNA Extraction Kit (TaKaRa Biotechnology). The mitochondrial genomes of matrilineal relatives (III-4, II-6, and II-8) were amplified by polymerase chain reaction (PCR) and sequenced as described elsewhere [14]. Each PCR product was purified and analyzed through direct sequencing on an ABI 3700 automated DNA sequencer. The results were compared to the revised Cambridge reference sequence (rCRS, GenBank Accession Number NC_012921.1) to analyze the mtDNA mutations [15].

### 2.4. Structural Analysis

The secondary structures of mt-tRNA^Ser(AGY)^ and tRNA^Cys^ were defined according to the published mt-tRNA molecules, based on the previous literature [16].

### 2.5. Conservation Assessments

To analyze the pathogenicity of m.12265A>G and m.5821G>A mutations, we used the ClustalW program (http://www.clustal.org) to predict the conservation of sequence among various species [17]. The conservation index (CI) was then used to assess the potential pathogenicity of these mutations, based on the previous investigation [18].

### 2.6. Generation of Transmitochondrial Cybrid Cell Lines

Cybrid cell lines were generated by fusion of *ρ*^0^ 206 cells with platelets [19], which were derived from the blood samples of the family members (III-4, II-6, and II-8) carrying the m.12265A>G and m.5821G>A mutations, as well as three genetically unrelated Chinese controls (C1, C2, and C3) which belonged to the same mitochondrial haplogroup D4a but without these mt-tRNA mutations [20]. Single cybrid clones were cultured in high-glucose DMEM medium with 10% FBS, without uridine or sodium pyruvate supplementation.

To see whether the cybrid cells were successfully established, PCR and direct sequencing were used to detect the presence of m.12265A>G and m.5821G>A mutations. The primers for detecting the m.12265A>G mutation were as follows: forward 5⁣′-TAT CAC TCT CCT ACT TAC AG-3⁣′; reversed 5⁣′-AGA AGG TTA TAA TTC CTA CG-3⁣′; the primers for detection of the m.5821G>A mutation were as follows: forward 5⁣′-CTA ACC GGC TTT TTG CCC-3⁣′; reversed 5⁣′-ACC TAG AAG GTT GCC TGG CT-3⁣′. The PCR fragments spanning mt-tRNA^Ser(AGY)^ and mt-tRNA^Cys^ genes were purified and subsequently sequenced in an ABI PRISM 3700 DNA Analyzer; the data were compared to rCRS results to detect the occurrence of the m.12265A>G and m.5821G>A mutations (GenBank Accession Number NC_012921.1) [15].

### 2.7. Analyses of Mitochondrial ATP, Membrane Potential (MMP), ROS, NAD^+^/NADH Ratio, Calcium Ion (Ca^2+^), and Extracellular Lactate Levels in Cybrid Cells

ATP production in the control and mutant cells was measured using an ATP bioluminescent somatic cell kit (Sigma-Aldrich) [21], while MMP was determined by using the JC-10 Assay Kit (Abcam), based on the manufacturer's instructions [22]. ROS was assessed with the MitoSOX reagent (Thermo Fisher Scientific), in accordance with the manufacturer's protocol [23]. The ratio of NAD^+^/NADH was determined according to the NAD^+^/NADH Assay Kit (Abcam) [24]. In addition, to analyze the Ca^2+^ concentrations, cells with and without mt-tRNA mutations were first incubated in 4 *μ*M Rhod 2-AM (Abcam) solution, and fluorescence was analyzed using a microplate reader (Molecular Devices) [25]. Extracellular lactate levels were detected using the Amplite Fluorometric L-Lactate Assay Kit (AAT Bioquest, United States), as previously described [26].

### 2.8. Quantitative PCR (qPCR) Analysis

Total RNA was isolated from six cybrids using Trizol reagent (Invitrogen), following the manufacturer's instructions, and 1000 ng of RNA was used with a reverse transcription kit (Vazyme). Mitochondrial transcription factor A (TFAM), a gene critical for mtDNA transcription [27], its mRNA expression was analyzed by qPCR using SYBR Green Master Mix (Transgene) based on the 2^−*ΔΔ*Ct^ method [28]. In addition, mtDNA copy number was determined using qPCR, according to the protocol described in our previous study [29]. The primers for *β*-actin were as follows: forward 5⁣′-CAC CAT TGG CAA TGA GCG GTT C-3⁣′, reversed 5⁣′-AGG TCT TTG CGG ATG TCC ACG T-3⁣′; the primers for TFAM were as follows: forward 5⁣′-AAC CAA AAA GAC CTC GTT CAG C-3⁣′; reversed 5⁣′-TTC AGC TTT TCC TGC GGT GA-3⁣′; the primers for mtDNA were as follows: forward 5⁣′-AAC ATA CCC ATG GCC AAC CT-3⁣′; reversed 5⁣′-AGC GAA GGG TTG TAG TAG CCC-3⁣′.

### 2.9. Analysis of OXPHOS Enzyme Activities

Mitochondria were first isolated from all cybrid cell lines based on the protocol as described previously [30]. The protein concentrations of the mitochondrial suspension were analyzed by a BCA Protein Assay Kit (Thermo Fisher). Enzymatic activity of Complexes I~IV and citrate synthase (CS) in control and mutant cybrids was assessed according to a protocol described [31]. The specific enzyme activities were normalized by CS activity.

### 2.10. Statistical Analyses

The SPSS 22.0 and GraphPad Prism 8.0.2 software were used to perform the statistical analyses. A *t*-test was used to measure the values of the two groups. Statistical significance was defined at *p* < 0.05.

## 3. Results

### 3.1. Clinical Features of the DCM Pedigree

As shown in Figure 1a, six of nine matrilineal relatives from this pedigree were affected by DCM, suggesting a maternal transmission. The clinical characteristics of the present four members of this pedigree are illustrated in Table 1 and Figure 1b; the LVEDd of the maternal individuals ranged from 5.26 to 6.73 cm, with an average of 5.82 cm, which was much thicker than the healthy subject (III-1). In addition, the average LVFS (23.8%) and LVEF (41.7%) in affected subjects were lower than those in controls.

The proband (III-4), aged 44, came from Hangzhou City of Zhejiang Province, visiting Hangzhou First People's Hospital for regular treatment of DCM. A comprehensive family history, physical assessments, and laboratory evaluations showed that none of these individuals, except for the proband's mother (II-8), had any other clinical diseases (hypertension, blood pressure: 140/70 mmHg).

### 3.2. Genetic Analysis

**Figure 2:**
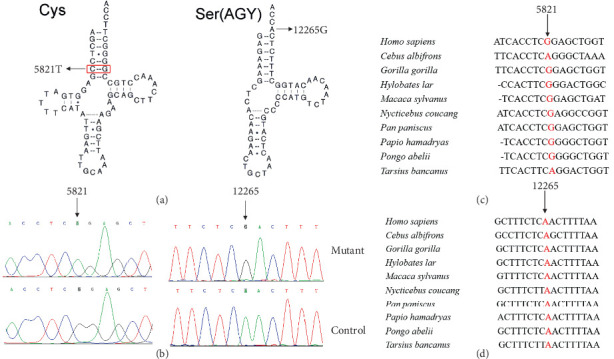
Characterization of DCM-related mt-tRNA^Cys^ 5821G>A and mt-tRNA^Ser(AGY)^ 12265A>G mutations. (a) Secondary structure of mt-tRNA^Cys^ and mt-tRNA^Ser(AGY)^, arrows indicate the m.5821G>A and m.12265A>G mutations. (b) Sanger sequence analyses of m.5821G>A and m.12265A>G mutations. (c) Conservation assessments of m.5821G>A mutation. (d) Conservation assessment of m.12265A>G mutation.

Since the DCM is maternally inherited, suggesting the mitochondrial involvement, we thus performed PCR amplification of the entire mtDNA genes from matrilineal relatives (II-6, II-8, and III-4) and 420 controls using 24 primers. Compared with the rCRS, we were able to identify a set of genetic polymorphisms that can be defined as mtDNA haplogroup D4a (Table 2), including five variants in the D-loop, three variants in the 12S rRNA, and two variants in the 16S rRNA and two mutations in the tRNAs (Figure 2a,b), while the remaining variants were mainly located in OXPHOS complexes. In addition, we identified several missense mutations: *ND1* 4216T>C (p.Tyr304His), *ND2* 5178C>A (p.Leu237Met), *A6* 8414C>T (p.Leu17Phe), 8584G>A (p.Ala20Thr), 8701A>G (p.Thr59Ala), 8860A>G (p.Thr112Ala), *ND3* 10398A>G (p.Thr114Ala), *ND6* 14766C>T (p.Thr7Ile), and *CytB* 15326A>G (p.Thr194Ala). These missense mutations were also examined by phylogenetic conservation assessments in various species including mouse [32], bovine [33], and *Xenopus laevis* [34]. Multialignment analysis suggested that the m.5821G>A and m.12265A>G mutations were highly conserved among the different species (Figure 2c,d), whereas the other variants were not evolutionary conserved. Furthermore, the m.5821G>A and m.12265A>G mutations were not detected in 420 control subjects, indicating that they may be involved in DCM progression.

Figure 2a reveals that the m.5821G>A mutation occurred at Position 6 in the acceptor arm of mt-tRNA^Cys^, disrupting a conserved base pairing (6C-67G), and may play a putative role in mt-tRNA structure and function. Furthermore, the novel m.12265A>G mutation was located at 3⁣′-end of mt-tRNA^Ser(AGY)^ acceptor arm (conventional Position 73), which was critical for the terminal cytosine–cytosine–adenosine (CCA) addition by tRNA nucleotidyl transferase 1 (TRNT1). Importantly, this enzyme was also necessary for tRNA aminoacylation [35]. Therefore, it was proposed that the mutation m.12265A>G may impair mt-tRNA^Ser(AGY)^ metabolism via affecting the maturation of this mt-tRNA.

### 3.3. Mitochondrial Functions Were Impaired in Mutant Cybrids

**Figure 3:**
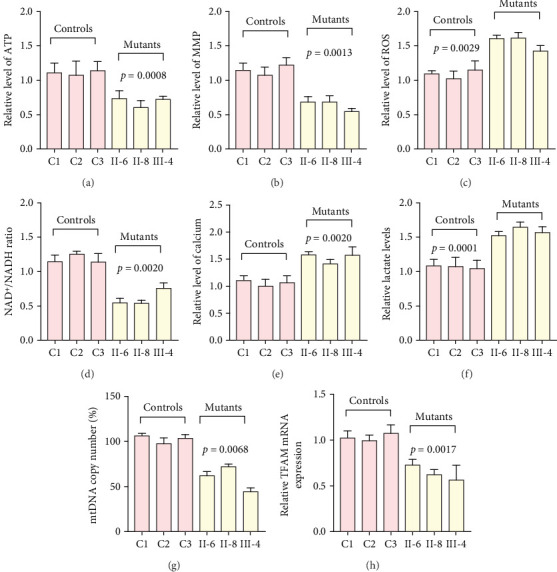
Analyses of mitochondrial functions in control and mutant cell lines. (a) ATP analysis; (b) MMP analysis; (c) ROS analysis; (d) NAD^+^/NADH ratio; (e) Ca^2+^ analysis; (f) lactate analysis; (g) mtDNA copy number analysis; (h) TFAM mRNA expression analysis. The data are expressed as the mean ± SD with three independent experiments.

**Figure 4:**
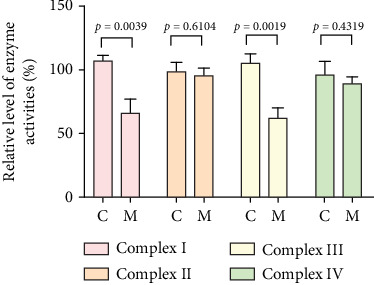
Analysis of OXPHOS enzymatic activities in mutant and control cybrids. C: control cells; M: mutant cells. The data are expressed as the mean ± SD with three independent experiments.

To further assess the impact of the m.5821G>A and m.12265A>G mutations on mitochondrial function, we measured the relative levels of mitochondrial ATP, MMP, ROS, NAD^+^/NADH ratio, Ca^2+^, lactate, mtDNA copy number, and TFAM mRNA expression in control and mutant cell lines. As shown in Figure 3, the mutant cells exhibited lower levels of ATP, MMP, NAD^+^/NADH ratio, mtDNA copy number, and TFAM mRNA expression than the control cells (*p* < 0.05). However, a significant increase in ROS, Ca^2+^, and lactate levels was observed in the mutant cybrid cells (*p* < 0.05). Furthermore, the Complex I and III enzyme activities were markedly decreased in mutant cybrids (*p* = 0.0039 and *p* = 0.0019, respectively), while the activities of Complex II and IV showed no statistically significance between control and mutant cell lines (Figure 4). Together, our data indicate that OXPHOS function is severely damaged in cybrids with m.5821G>A and m.12265A>G mutations.

## 4. Discussion

The myocardium requires a high amount of aerobic metabolism in order to provide blood and energy substrates to all body organs. Mitochondria play a critical role in ATP production and maintaining the bioenergetic functions of cardiovascular systems [36]. Specifically, OXPHOS generated more than 90% of ATP required by cardiac cells to keep their normal functions. In addition, mitochondria play an important role in buffering cytosolic Ca^2+^ and regulating apoptosis via the mitochondrial permeability transition pore (mPTP) [37]. Mutations in mtDNA lead to disorders in the OXPHOS system and defects in the synthesis of cardiac muscle contraction proteins [38]. As a result, it causes insufficient generation of mitochondrial ATP, enhanced metabolic requirements for ATP by the myocardium, and the accumulation of ROS, resulting in damage to myocardial cells, eventually inducing DCM [39].

In the present study, we have performed clinical, genetics, and molecular characterizations of a three-generation Chinese family with DCM. Notably, the DCM subjects exhibited a wide variety of ages at onset, ranging from 44 to 72 years, with an average of 55 years. Furthermore, the LVEDd ranged from 5.26 to 6.73 cm, while the LVFS ranged from 15% to 29.7%; it should be noted that the proband's sister (III-3), who also carried the m.12265A>G and m.5821G>A mutations, had normal clinical presentations and but cannot exclude the possibility of developing DCM phenotype over time.

Interestingly, among nine matrilineal relatives, six suffered from DCM (two males/four females); the ratio of male to female was 1:2, suggesting that in this pedigree, women seemed to be more prone to develop DCM than men despite that they harbored the same mtDNA pathogenic mutations. Although previous clinical studies revealed that DCM had a sex ratio of around 1.9: 1 men to women in the Chinese population [40], its detailed molecular mechanism was not fully elucidated. The possible reasons can be listed as follows: first, as DCM was an important risk factor for heart failure, epidemiological data demonstrated that women were about twice as likely to develop heart failure compared to men [41]. Second, DCM can be divided into genetic and nongenetic categories, and environmental risk factors also contribute to play putative roles in this disease [42]. Thus, other unidentified environmental and genetic risk factors, or even personal lifestyles such as dietary habits, smoking, and unusual sleeping times, intended to interact with the m.12265A>G and m.5821G>A mutations, enhanced the expressivity of female patients to develop DCM in this pedigree.

Maternal transmission of DCM in this pedigree suggests that mtDNA mutations are the important contributors to this disorder. Mutation analysis revealed the presence of a novel mt-tRNA^Ser(AGY)^ 12265A>G mutation that had not been found in any published mitochondrial research databases from Mitomap (http://www.mitomap.org), mtDB (http://www.genpat.uu.se/mtDB), and mtSNP (http://mtsnp.tmig.or.jp). In fact, the highly conserved m.12265A>G mutation occurred at Position 73 in the 3⁣′-end of mt-tRNA^Ser(AGY)^; interestingly, the m.5587T>C, which was located at the same position as mt-tRNA^Ala^, was found to impair the 3⁣′-end processing of mt-tRNA^Ala^ precursors catalyzed by RNase Z and inhibit the CCA addition by TRNT1 [43]. Importantly, the addition of CCA is believed to stabilize tRNA selection, playing an indispensable role in mt-tRNA quality control; hence, we believe that the novel m.12265A>G, which is similar to the m.5587T>C mutation, may cause aberrant 3⁣′-end processing of mt-tRNA^Ser(AGY)^ and impair mt-tRNA maturation and protein translation [44].

While 5821G>A in mt-tRNA^Cys^ disrupts Watson–Crick base pairing (6C-67G), which is extremely conserved from different species [16], note that the m.5650G>A mutation occurring at the same position as mt-tRNA^Ala^ had been reported to cause mitochondrial dysfunction in pure myopathy [45]. Moreover, the m.5821G>A mutation resides at the acceptor arm of mt-tRNA^Cys^; mutation at this position in mt-tRNA has been shown to play important roles in recognition by its cognate aminoacyl-tRNA synthetases (ARSs) [46], an enzyme that is critical for protein synthesis [47]. Thus, we propose that the m.5821G>A mutation affects the aminoacylation ability of mt-tRNA^Cys^, impairing mitochondrial protein synthesis.

As mitochondrial functions/dysfunctions are tightly regulated by both nuclear DNA (nDNA) and mtDNA, they make it difficult to assess whether nDNA or mtDNA is responsible for the OXPHOS system deficiency. Cybrid is an ideal cell model to solve this problem because the noise from nuclear background can be kept constant [48]. In this study, we observed that cybrid cells with m.12265A>G and m.5821G>A mutations caused a defect in OXPHOS function; mitochondrial ATP, MMP, and NAD^+^/NADH ratio were markedly decreased, while the relative levels of ROS, Ca^2+^, and extracellular lactate were increased significantly. Indeed, MMP is an important component in ATP storage during OXPHOS processing. MMP also plays a critical role in the maintenance of mitochondrial homeostasis and is a reliable indicator of apoptosis [49]. The NAD, a coenzyme found in every cell which is essential for energy production, exists in two distinct forms, called NAD^+^ (oxidized) and NADH (reduced), and the reduced NAD^+^/NADH ratio is believed to affect cellular energy metabolism, suggesting an impairment of cellular function and viability [50]. Under physiological conditions, a slight increase in Ca^2+^ concentration may lead to the activation of several enzymes involved in the tricarboxylic acid (TCA) cycle, enhancing ATP synthesis and promoting metabolic adaptation [51]. However, overloaded Ca^2+^ in cardiomyocytes increases the sensitivity of organelles to proapoptotic stimuli, decreasing the threshold for mPTP opening and resulting in apoptosis [52]. Importantly, lactate, a metabolic waste product generated from glycolysis, is regarded as a myocardial fuel that can be converted into pyruvate via the enzyme lactate dehydrogenase (LDH), thereby feeding the TCA cycle to generate ATP [53]. Furthermore, significant reductions in mtDNA copy number, as well as TFAM mRNA expression, together with the reduced Complex I and III enzymatic activities suggested the presence of OXPHOS is exhibited in cybrids with the m.12265A>G and m.5821G>A mutations.

Our study has several limitations; first of all, because DCM-associated mtDNA mutations are tissue-specific, it is necessary to perform molecular and biochemical analyses of muscle biopsy from participants. The second limitation is the relatively small sample size enrolled in this study. Future research may involve investigating the mechanisms underlying the observed associations or large-scale studies to confirm the current findings.

## 5. Conclusions

In conclusion, this is the first report concerning the molecular pathophysiology of the mt-tRNA^Ser(AGY)^ 12265A>G and mt-tRNA^Cys^ 5821G>A mutations associated with DCM. Our study supports a positive link between novel mtDNA mutations and DCM progression and, thus, targeted aggressive management for high-risk patients. The homoplasmic forms of mtDNA mutations and phenotypic variability of DCM in this pedigree suggest that other modified factors, including environmental, personal lifestyle, and epigenetic modifications, may contribute to the development of DCM in individuals carrying the mt-tRNA^Ser(AGY)^ 12265A>G and mt-tRNA^Cys^ 5821G>A mutations.

## Figures and Tables

**Table 1:** Summary of the clinical data for members of the Chinese pedigree with DCM.

**Subjects**	**Sex**	**Age at onset (year)**	**Age at test (year)**	**BP (mmHg)**	**LVEDd (cm)**	**LVFS (%)**	**LVEF (%)**	**ECG rhythm**	**Abnormal Q wave**	**ST-T change**
II-6	Female	66	72	110/70	5.48	26.8	51.8	Sinus	+	+
II-8	Female	59	63	140/70	6.73	15.0	31.0	Atrial fibrillation	+	+
III-4	Male	44	44	133/86	5.26	29.7	42.49	Atrial fibrillation	−	+
III-1	Female	/	40	126/75	4.54	33.4	62.2	Sinus	−	−

Abbreviations: BP, blood pressure; LVEDd, left ventricular end diastolic diameter; LVEF, left ventricular ejection fraction; LVFS, left ventricular fractional shortening.

**Table 2:** mtDNA variants of matrilineal relatives in Chinese pedigree with DCM.

**Gene**	**Position**	**Replacement**	**Amino acid change**	**rCRS** ^**a**^	**Conservation in H/B/M/X** ^**b**^	**Previously reported** ^**c**^
D-loop	73	A to G		A		Yes
	263	A to G		A		Yes
	310	InsC		T		Yes
	16132	T to C		T		Yes
	16224	T to C		T		Yes

12S rRNA	750	A to G		A	A/A/A/–	Yes
	1107	T to C		T	T/C/T/T	Yes
	1438	A to G		A	A/A/A/G	Yes

16S rRNA	2706	A to G		A	A/G/A/A	Yes
	3107	Del N		N	N/T/T/–	Yes

*ND1*	4200	A to T		A		Yes
	4216	T to C	Tyr to His	T	Y/Y/H/H	Yes

*ND2*	5178	C to A	Leu to Met	C	L/T/T/T	Yes

tRNA^Cys^	5821	G to A		G	G/G/A/G	Yes

*CO1*	7028	C to T		C		Yes

*A6*	8414	C to T	Leu to Phe	C	L/F/M/W	Yes
	8584	G to A	Ala to Thr	G	A/V/V/I	Yes
	8701	A to G	Thr to Ala	A	T/S/L/Q	Yes
	8860	A to G	Thr to Ala	A	T/A/A/T	Yes

*CO3*	9540	T to C		T		Yes
	9545	A to G		A		Yes

*ND3*	10397	A to G		A		Yes
	10398	A to G	Thr to Ala	A	T/T/T/A	Yes
	10400	C to T		C		Yes

*ND4*	11722	G to A		G		Yes
	11917	G to A		G		Yes

tRNA^Ser(AGY)^	12265	A to G		A	A/A/A/A	No

*ND5*	12705	C to T		C		Yes

*ND6*	14318	T to C		T		Yes
	14766	C to T	Thr to Ile	C	T/S/T/S	Yes

*CytB*	14927	A to G		A		Yes
	15043	G to A		G		Yes
	15301	G to A		G		Yes
	15326	A to G	Thr to Ala	A	T/M/I/I	Yes

^a^rCRS: revised Cambridge reference sequence.

^b^Conservation of amino acid for polypeptides or nucleotide for rRNAs in human (H), bovine (B), mouse (M), and *Xenopus laevis* (X).

^c^See the online mitochondrial genome database Mitomap (http://www.mitomap.org), mtDB (http://www.genpat.uu.se/mtDB), and mtSNP (http://mtsnp.tmig.or.jp).

## Data Availability

The raw data supporting the conclusions of this article will be made available by the authors upon request.
